# Nephroprotective Effects of Benzyl Isothiocyanate and Resveratrol Against Cisplatin-Induced Oxidative Stress and Inflammation

**DOI:** 10.3389/fphar.2018.01268

**Published:** 2018-11-21

**Authors:** Abdelazim Ibrahim, Fahad A. Al-Hizab, Abdelrahman Ibrahim Abushouk, Mohamed M. Abdel-Daim

**Affiliations:** ^1^Department of Pathology, College of Veterinary Medicine, King Faisal University, Al-Hasa, Saudi Arabia; ^2^Department of Pathology, College of Veterinary Medicine, Suez Canal University, Ismailia, Egypt; ^3^Faculty of Medicine, Ain Shams University, Cairo, Egypt; ^4^Department of Pharmacology, Faculty of Veterinary Medicine, Suez Canal University, Ismailia, Egypt

**Keywords:** benzyl isothiocyanate, cisplatin, kidney, resveratrol, mice

## Abstract

This study was performed to compare the nephroprotective effects of benzyl isothiocyanate (BITC) and resveratrol (RES) and investigate the nephroprotective efficacy of their combination against cisplatin-induced acute renal injury. Five animal groups (each of eight) received either normal saline, a single intraperitoneal injection of cisplatin (20 mg/kg) at the sixth day, cisplatin plus oral RES (30 mg/kg) or BITC (100 mg/kg in diet), or a combination of both for 10 days. Compared to saline-treated mice, cisplatin-intoxicated mice had significantly higher (*p* < 0.05) serum levels of urea, creatinine, interleukin-1β (IL-1β), and tumor necrosis factor-α. Moreover, biochemical analysis of kidney tissue homogenates showed that cisplatin intoxication was associated with significantly higher (*p* < 0.05) tissue levels of malondialdehyde (MDA) and lower levels of reduced glutathione and activities of endogenous antioxidant enzymes (glutathione peroxidase, superoxide dismutase, and catalase) in comparison to normal controls. Histopathological and immunohistochemical examinations of renal tissue slices from cisplatin-intoxicated mice showed interstitial leukocytic infiltration, tortuous tubules with vacuolated epithelium, luminal casts, and overexpression of cyclooxygenase-II enzyme. On the other hand, treatment with RES or BITC ameliorated all the previous parameters. The effects of both compounds were comparable in all assessed parameters, except IL-1β serum concentration and renal tissue MDA concentration (which were significantly lower in the RES group). Interestingly, treatment with BITC and RES combination restored the normal concentrations of all the aforementioned biochemical parameters, as well as near normal histological and immunohistochemical pictures. In conclusion, BITC exerted nearly comparable nephroprotective, antioxidant, and anti-inflammatory effects to RES and the combination of both agents showed more potent nephroprotective effects against cisplatin than each one alone.

## Introduction

**FIGURE 1:**
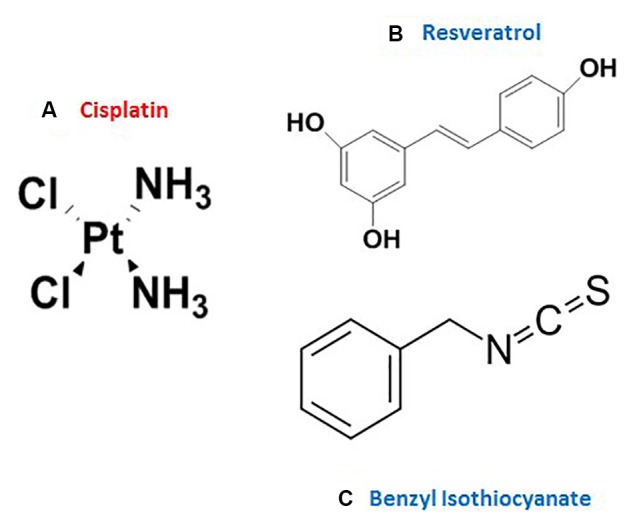
The chemical structure of **(A)** cisplatin, **(B)** resveratrol, and **(C)** benzyl isothiocyanate.

Cisplatin (*Cis*-diamminedichloroplatinum) is a potent chemotherapeutic drug (Figure 1A), used in the management of several malignancies, such as advanced bladder carcinoma and metastatic ovarian and testicular tumors (Galanski, 2006). However, its clinical use is restricted due to tumor resistance and major dose-related side effects, such as nephrotoxicity, ototoxicity, and bone marrow depression (Arany and Safirstein, 2003; Sastry and Kellie, 2005; Dasari and Tchounwou, 2014). With the early clinical application of cisplatin, more than 50% of patients reported dose-related renal insufficiency (Goldstein and Mayor, 1983); however, using saline hydration and diuresis during cisplatin administration reduced these rates to about 20% (Hartmann et al., 1999). Among the several mechanisms that were proposed for cisplatin-induced renal damage, the oxidative stress-mediated cytotoxicity has been prominent. Cisplatin induces mitochondrial dysfunction through inhibition of the mitochondrial complexes (I–IV), increasing the production of reactive oxygen species (ROS) (Kruidering et al., 1997). Moreover, cisplatin directly interacts with glutathione (GSH), suppressing its antioxidant effect (Arany and Safirstein, 2003; Siddik, 2003).

Resveratrol is a polyphenolic phytoalexin (present naturally in grapes and berries) (Figure 1B) that possesses antioxidant, anti-infective, and cardioprotective effects (Frémont, 2000; Catalgol et al., 2012). Previous studies have revealed the renoprotective effects of RES against diabetic nephropathy (Sharma et al., 2006; Palsamy and Subramanian, 2011), renal injuries induced by aldosterone (Yuan et al., 2012), ischemia–reperfusion and sepsis (Giovannini et al., 2001; Holthoff et al., 2012), as well as drug-related nephrotoxicity (Miller et al., 2010). Resveratrol (RES) exerts its cytoprotective effect by scavenging ROS, including the hydroxyl, superoxide, and peroxynitrite radicals (Leonard et al., 2003; Holthoff et al., 2010). Moreover, it modulates the expression and activities of endogenous antioxidant enzymes like superoxide dismutase (SOD), catalase (CAT), and glutathione peroxidase (GPx) (Mokni et al., 2007; Robb et al., 2008; Kitada et al., 2011).

Benzyl isothiocyanate (BITC) is a natural compound (Figure 1C), present in cruciferous vegetables (Miyoshi et al., 2004). In a study to investigate the chemopreventive effects of ITCs, BITC significantly ameliorated the oxidative burst by inhibiting the leukocytic NADPH oxidase, which produces large amounts of superoxide radicals (Miyoshi et al., 2004). Moreover, BITC has a chemosensitizing activity, which improves the efficacy of cisplatin chemotherapy (Wattenberg, 1987; Morse et al., 1993). This is probably because it induces apoptosis in tumor cells through the activation of mitogen-activated protein kinases (MAPKs) and inhibition of the extracellular signal-regulated kinase (Lee et al., 2012).

To our knowledge, there is a lack of published data over the chemoprotective effect of BITC against cisplatin-induced nephrotoxicity. Therefore, the aims of this experiment were to investigate the renoprotective effects of BITC, to compare its efficacy to the established antioxidant (RES), and to assess the efficacy of BITC–RES combination against cisplatin-induced nephrotoxicity in mice.

## Materials and Methods

### Chemicals

Cisplatin (1 mg/ml vial) in a clinical formulation and BITC were obtained from Sigma (St. Louis, MO, United States), while RES was bought from NOW Foods (IL, United States). All biochemical analysis kits were purchased from Biodiagnostics Company (Dokki, Giza, Egypt) except tumor necrosis factor-α (TNF-α) (BioSource International, Camarillo, CA, United States) and interleukin-1β (IL-1β) kits (Glory Science Ltd., Del Rio, TX, United States). All other chemicals were of high analytical grades.

### Animals and Experimental Design

Forty Swiss Albino mice (males, weight 22 ± 5 g) were obtained from the Egyptian Company for Biological Products and Vaccines. Animals were acclimatized in a well-ventilated room with normal light/dark (12 h each) cycle and temperature (25 ± 2°C) and were supplied with adequate food and water. The animal handling procedures in this study were approved by the local Research Ethical Committee at Suez Canal University, Egypt (Approval No.: 201513).

**FIGURE 2:**
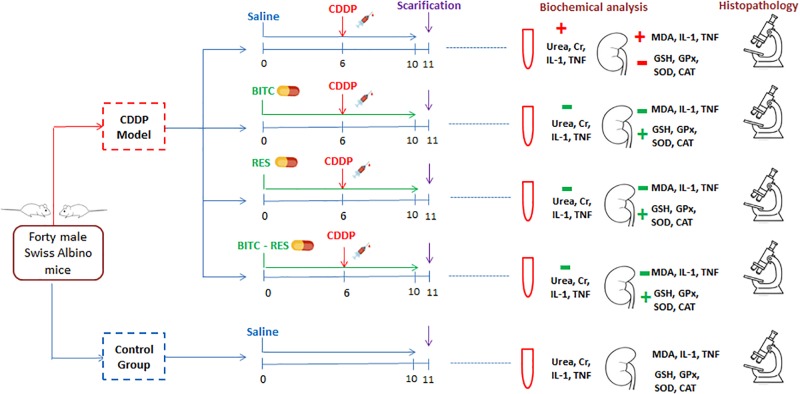
A flow diagram of animal allocation, treatment regimens, and outcome assessment. BITC: benzyl isothiocyanate, CAT: catalase, GPx: glutathione peroxidase, GSH: glutathione, IL-1β: interleukin-1β, MDA: malondialdehyde, RES: resveratrol, SOD: superoxide dismutase, and TNF: tumor necrosis factor.

Mice were randomly allocated into five groups (*n* = 8 per group). Group I mice received normal saline and acted as a negative control group. Mice in the other four groups received a single intraperitoneal (i.p.) dose of cisplatin (20 mg/kg) at the sixth day of the experiment (Ueki et al., 2013). Moreover, mice in group III [cisplatin (CDDP)–BITC] received daily oral supplementation with BITC (100 mg/kg in diet) for 10 days (Kim et al., 2015) and mice in group IV (CDDP–RES) received daily oral supplementation with RES (30 mg/kg) for the same duration (Zhou et al., 2015). Mice in group V (CDDP–BITC–RES) received a combination of BITC and RES for 10 days (at the same aforementioned doses) (Figure 2).

### Biochemical Analysis

On the 11th day, blood samples were obtained under isoflurane anesthesia. After clotting at 25°C, samples were centrifuged for 15 min (at 3000 rpm) and the resulting sera were stored at −20°C until used to assess the serum concentrations of creatinine and urea, according to the methods by Larsen (1972) and Coulombe and Favreau (1963), respectively.

Later, mice were sacrificed and tissue pieces of the mice kidneys were removed, washed with saline, and homogenized in potassium phosphate buffer. Then, the homogenate underwent two cycles of centrifugation (at 600 and 10,000 × *g*) and supernatants were utilized to measure the concentrations of MDA [lipid peroxidation marker according to Uchiyama and Mihara (Uchiyama and Mihara, 1978)], GSH (according to Beutler et al., 1963), GPx (according to Paglia and Valentine, 1967), SOD (according to Nishikimi et al., 1972), and CAT (according to Aebi, 1984). The serum and tissue concentrations of IL-1β and TNF-α were measured as per the manufacturer’s instructions and the absorbance was read by an automated ELISA reader at 420 nm.

### Histopathology and Immunohistochemistry

Parts of the removed mice kidneys were fixed in 10% buffered formalin and were then embedded into paraffin blocks. Then, 4-μm-thick sections were cut and stained with hematoxylin and eosin (H&E) and examined with a light microscope. Histopathological alterations were evaluated using a semiqunatitative scale. Renal sections were examined blindly (10 fields per slide) at 400× magnification. The used scoring system was (0: normal, 1: <10%, 2: 11–25%, 3: 26–45%, 4: 46–75%, 5: >76%) for the percentage of epithelial cell necrosis, tubular cast, degree of interstitial inflammation, and tubular dilation.

For immunohistochemical examination, paraffin-embedded renal sections were de-paraffinized (in xylene) and rehydrated in alcohol, followed by antigen unmasking in citrate buffer (pH 6.0); 5% normal goat or rabbit serum in phosphate-buffered saline was used to block non-specific binding sites. Later, sections were incubated with primary antibodies against Cox-II (1:100), followed by specific biotin-conjugated secondary antibodies (Vector Laboratories, Burlingame, CA, United States). For color development, Vector-ABC streptavidin-peroxidase kits were added to the slides and sections were counterstained with diluted hematoxylin. For quantification, positively stained cells (brown-colored) were identified using the double color thresholding of ImageJ program.

### Data Analysis

All obtained values were expressed as means ± standard deviations (SD), calculated by SPSS (version 22, Chicago, IL, United States). The significance of between-group differences was tested using the one-way Analysis of Variance, followed by the *post hoc* Tukey’s test. The results of all groups were evaluated in comparison to the control and CDDP groups. Moreover, the results of the CDDP–BITC group were compared to those of the CDDP–RES and CDDP–BITC–RES groups. The significance of these differences was assigned at a *p*-value of <0.05.

## Results

### Biochemical Serum Analysis

**FIGURE 3:**
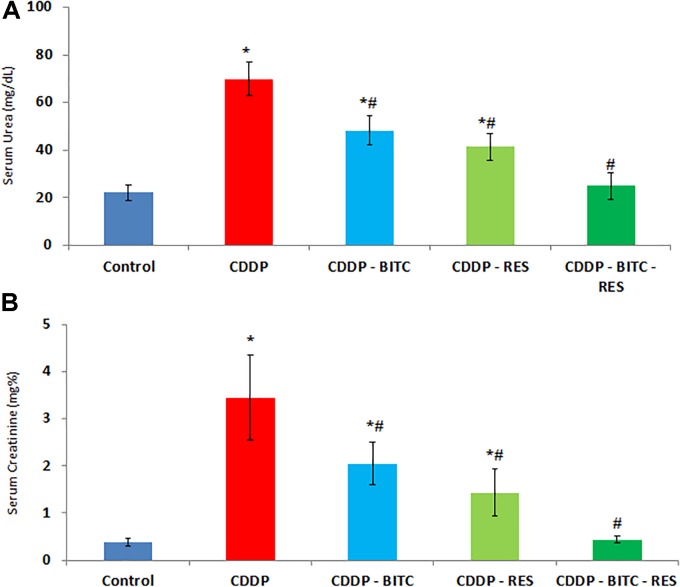
The effects of RES and BITC treatment on serum concentrations of **(A)** urea and **(B)** creatinine in cisplatin-intoxicated mice. Data are means ± standard deviations. CDDP, cisplatin. ^∗^Significantly different from the normal control group at *p* < 0.05. ^#^Significantly different from the CDDP group at *p* < 0.05.

**FIGURE 4:**
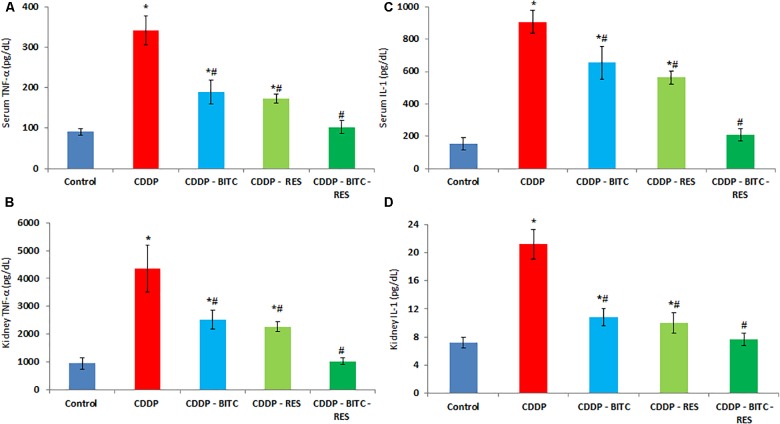
The effects of RES and BITC treatment on serum and renal tissue levels of **(A,B)** tumor necrosis factor-α **(C,D)** interleukin-1β in cisplatin-intoxicated mice. Data are means ± standard deviations. CDDP, cisplatin. ^∗^Significantly different from the normal control group at *p* < 0.05. ^#^Significantly different from the CDDP group at *p* < 0.05. Additionally, note that serum IL-1β concentration in CDDP–RES group is significantly lower than in the CDDP–BITC group (*p* < 0.05).

Compared to normal control mice, cisplatin-intoxicated mice showed significantly (*p* < 0.05) higher serum levels of urea, creatinine (Figure 3), TNF-α and IL-1β (Figures 4A,C). However, cisplatin-intoxicated mice, treated by RES and BITC, alone or in combination, had significantly lower (*p* < 0.05) serum levels of the aforementioned parameters than those treated with cisplatin alone. The effects of both compounds were comparable in all assessed parameters, except IL-1β serum concentration (that was significantly lower in the RES-treated group). Interestingly, the serum concentrations of these parameters were restored to the normal ranges in the combination treatment group.

### Biochemical Tissue Analysis

Cisplatin-intoxicated mice exhibited significantly higher levels of MDA, TNF-α and IL-1β (Figures 4B,D), as well as significantly lower (*p* < 0.05) GSH concentration and enzymatic activities of GPx, SOD, and CAT in comparison to normal controls. On the other hand, cisplatin-intoxicated mice co-treated with RES or BITC or a combination of both showed significantly lower MDA, IL-1β, and TNF-α levels and significantly higher (*p* < 0.05) GSH concentration and antioxidant enzymatic activities. The effects of both compounds were comparable in all assessed parameters, except renal tissue MDA concentration (that was significantly lower in the RES-treated group). Of note, treatment with RES or RES plus BITC restored the normal tissue concentrations of some (GPx and CAT) or all aforementioned parameters, respectively (Table 1).

**Table 1:** The effects of RES and BITC treatment on renal tissue lipid peroxidation GSH concentration and activities of antioxidantenzymes in cisplatin-intoxicated mice.

	Control	CDDP	CDDP–BITC	CDDP–RES	CDDP–RES–BITC
MDA (nmol/g)	5.1 ± 0.78	14.9 ± 1.4^∗^	10.9 ± 1.45^∗#^	8.9 ± 1.22^∗#^	5.8 ± 1.04^#^
GSH (mmol/g)	13.6 ± 0.77	5.2 ± 0.75^∗^	10.7 ± 0.61^∗#^	11.24 ± 1.14^∗#^	13.4 ± 2.1^#^
GPx (mol/g)	6.92 ± 1.4	2.98 ± 0.63^∗^	4.9 ± 1.04^∗#^	5.6 ± 0.94^#^	6.6 ± 0.97^#^
SOD (U/g)	20.75 ± 4.14	7.2 ± 1.6^∗^	11.7 ± 1.9^∗#^	14.5 ± 2.1^∗#^	19.8 ± 2.6^#^
CAT (U/g)	4.35 ± 0.52	1.9 ± 0.55^∗^	3.03 ± 0.32^∗#^	3.54 ± 0.75^#^	4.17 ± 0.87^#^

*Data are means ± standard deviations. CDDP, cisplatin; GSH, glutathione. ^∗^Significantly different from the normal control group at p < 0.05. ^#^Significantly different from the CDDP group at p < 0.05. Note that the all parameters are comparable in CDDP–RES and CDDP–BITC groups, except for MDA, which is significantly lower in the CDDP–RES group (p < 0.05).*

### Histopathological Examination

**FIGURE 5:**
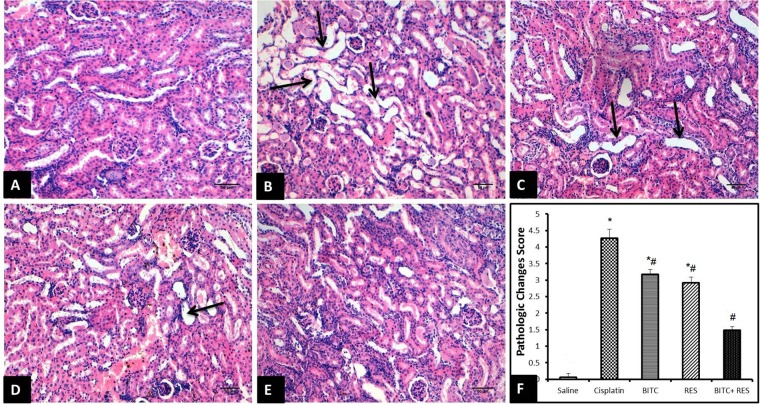
H&E sections of the kidney (Bar = 50 μm). **(A)** Saline-treated mice revealing normal kidney tissue morphology. **(B)** Cisplatin-treated mice revealing marked dilation and twisting of several renal tubules (arrows). **(C)** Slight improvement of renal tissue in CDDP–RES-treated mice (arrows). **(D)** Moderate histopathologic changes are seen in CDDP–BITC-treated mice (arrow). **(E)** Mice treated with BITC and RES combination showing mild morphologic changes. **(F)** Semiquantitative analysis of the histopathological changes (^∗^significantly different from the normal control group at *p* < 0.05, ^#^significantly different from the CDDP group at *p* < 0.05).

Control mice showed normal renal histology (Figure 5A), whereas those injected with cisplatin exhibited pathological alterations in about 40% of renal tubules, especially in the renal cortex and the corticomedullary junction. Affected tubules were ecstatic and tortuous. Their lining epithelium was attenuated or contained clear vacuoles within the cytoplasm. Some epithelial cells had hypereosinophilic cytoplasm with pyknotic nuclei (necrosis). The lumina of some tubules were filled with homogenous eosinophilic materials. Moreover, the interstitial tissue showed multifocal infiltration with moderate numbers of lymphocytes and macrophages (Figure 5B).

On the contrary, mice treated with RES (Figure 5C) or BITC (Figure 5D) alone showed moderate comparable degrees of the previously mentioned histopathological changes in about 25–30% of renal tubules. Interestingly, the renal tissue from mice that received both RES and BITC showed minimal pathological alterations in few scattered tubules (Figure 5E). Semiquantitative analysis showed that treatment with BITC or RES alone significantly reduced the renal tissue histopathological changes, compared to mice treated with cisplatin alone. This reduction was more significant in the BITC–RES combination group than in mice, treated with RES or BITC alone (Figure 5F).

### Immunohistochemical Examination

**FIGURE 6:**
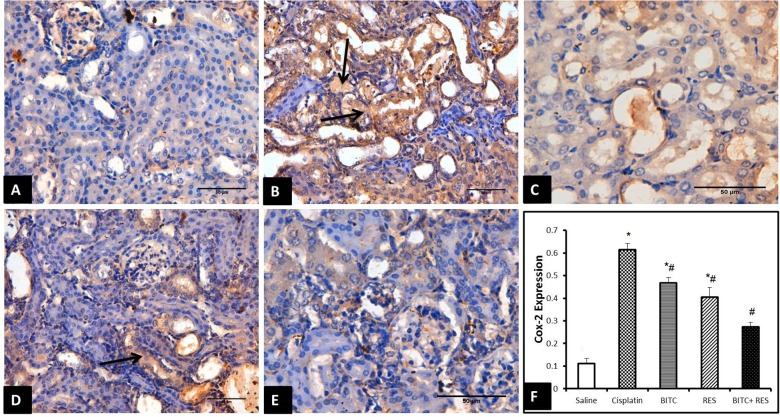
COX-II immunohistochemical examination (Bar = 50 μm) of renal tissue samples from **(A)** saline-treated mice showing minimal COX-II expression, **(B)** cisplatin-intoxicated mice revealing many Cox-II-positive epithelial cells (arrows), **(C)** cisplatin-intoxicated mice treated with RES and **(D)** cisplatin-intoxicated mice treated with BITC showing moderate reductions in COX-II-positive cells (arrow), and **(E)** cisplatin-intoxicated mice treated with a combination of RES and BITC showing minimal COX-II expression. **(F)** Quantitative Cox-II expression in the cytoplasm of renal epithelial cells (^∗^significantly different from the normal control group at *p* < 0.05, ^#^significantly different from the CDDP group at *p* < 0.05).

Cisplatin exposure in mice significantly increased the protein expression of COX-II enzyme within the cytoplasm of the tubular epithelial cells (Figure 6B) in comparison to the control group which revealed mild or absent immunoreactivity (Figure 6A). Cisplatin-intoxicated mice that received either RES (Figure 6C) or BITC (Figure 6D) showed comparable significant reductions in the expression of COX-II enzyme in comparison to mice treated with cisplatin alone. The Cox-II expression was significantly lower in the combination group (Figure 6E) than in mice, treated with RES or BITC alone (Figure 6F).

## Discussion

To our knowledge, this experiment is the first to examine the nephroprotective effects of BITC and RES combination against cisplatin-induced nephrotoxicity. The main findings in this study were that RES and BITC could improve the cisplatin-induced: (1) acute kidney injury (histological improvement and reduction of serum urea and creatinine levels), (2) inflammation (reduction of IL-1β and TNF-α levels in the serum and kidney, reduced COX-II expression in the renal tissue, and reduced leukocytic infiltration on histopathological examination), and (3) oxidative stress (reduced renal tissue MDA and NO levels and increased GSH concentration and enzymatic activities of SOD and CAT). Moreover, the nephroprotective effects in all investigated parameters were more significant with the BITC–RES combination than with either drug alone.

The mechanisms of cisplatin-induced nephrotoxicity are sophisticated; however, the induction of oxidative stress and inflammation remains the most established theory. In agreement with previous studies, cisplatin administration in laboratory animals induced oxidative stress and suppressed the endogenous antioxidant machinery (Miller et al., 2010; Topcu-Tarladacalisir et al., 2016). Moreover, we showed that cisplatin increases serum and tissue levels of TNF-α, which can be produced by several immune and non-immune cells, including the renal epithelium. TNF-α can induce direct toxicity to glomerular and tubular cells and activate the intrinsic and extrinsic apoptotic pathways (Ramesh and Reeves, 2002, 2003, 2004). In the same vein, the increased serum and tissue levels of IL-1β and the upregulated expression of COX-II increase the production of prostaglandin-E2 by renal mesangial cells, which changes the glomerular hemodynamics and filtration (Duran-Salgado and Rubio-Guerra, 2014) (as reflected on serum urea and creatinine in our data).

To minimize the nephrotoxic effects of cisplatin, we investigated whether BITC and RES, which are natural food-based compounds, can protect the renal tissues against cisplatin-induced nephrotoxicity. Our data revealed that RES mitigated cisplatin-induced oxidative stress and inflammation, confirming the findings of previous studies in that regard (Do Amaral et al., 2008; Valentovic et al., 2014; Osman et al., 2015). Although RES is established as an antioxidant, Kruidering et al. (1997) suggested that cisplatin cytotoxicity is not directly related to lipid peroxidation and that it induces ROS production due to leukocytic infiltration. Therefore, the anti-inflammatory effect of RES may be more important than its antioxidant role in this regard. Another study by Kim et al. (2015) attributed the nephroprotective effect of RES against cisplatin toxicity to increasing SIRT1 enzyme expression, which inhibits cisplatin-induced p53 apoptosis (Kim, 2011).

Likewise, our study showed that BITC exerted an antioxidant activity by ameliorating cisplatin-induced inflammation and upregulating the expression of antioxidant enzymes. Other studies showed that BITC can suppress inflammation-related carcinogenesis by suppressing superoxide radicals’ generation within inflammatory leukocytes, exposed to the pro-oxidant [12-*O*-tetradecanoylphorbol-13-acetate (TPA)] (Miyoshi et al., 2004; Nakamura et al., 2004). In addition, both agents (RES and BITC) have shown a chemosensitizing effect with cisplatin by increasing tumor cell uptake of cisplatin (Osman et al., 2015) or initiating apoptosis in tumor cells (Wattenberg, 1987; Morse et al., 1993). Therefore, their combination with cisplatin can offer extra protection and a more potent antimutagenic efficacy.

Comparing both agents (BITC and RES) showed that BITC exerted comparable nephroprotective, antioxidant, and anti-inflammatory effects to RES in most assessed parameters. However, the reductions in the serum IL-1β and renal tissue MDA concentrations were more significant in the RES-treated group, compared to the BITC-treated group. Moreover, RES could restore the normal renal tissue concentrations of GPx and CAT enzymes. Whether these few differences are due to additional molecular mechanisms for RES or could be negated by increasing BITC dose should be further investigated. Overall, the nephroprotective effects of both compounds were comparable.

Interestingly, the combination of RES and BITC showed significantly more potent antioxidant and anti-inflammatory activities than either agent alone. These findings indicate that BITC augments the antioxidant and anti-inflammatory effects of RES by increasing the expression of antioxidant enzymes and reducing the expression of pro-inflammatory cytokines. Further studies should investigate if other mechanisms are involved in BITC–RES combination nephroprotection. A limitation of this study is that the drug combination index (DCI) was not calculated to determine whether the effects of both agents are additive or synergistic (Chou and Talalay, 1983; Chou and Talalay, 1984). Future studies are encouraged to determine this relationship and test the drug combination in animal models with malignant tumors, receiving cisplatin chemotherapy.

Histopathological and immunohistochemical examinations confirmed the findings of our biochemical analysis. The observed cellular degeneration and tubular casts indicate impaired glomerular filtration and account for the recorded deteriorations in renal functions. Similarly, the increased leukocytic infiltration goes in parallel with the increased serum and tissue levels of TNF-α and IL-1β. Similar observations were recorded in former studies on cisplatin nephrotoxicity (Do Amaral et al., 2008; Topcu-Tarladacalisir et al., 2016). In contrast, concomitant treatment with BITC or RES significantly ameliorated all pathological changes and these effects were more marked in the combination group than in either agent groups. In agreement with previous studies, cisplatin-induced renal injury is associated with increased COX-II expression (Jia et al., 2011; Yu et al., 2017). Besides synthesizing pro-inflammatory cytokines, COX-II is involved in the production of vasoconstrictor thromboxanes, leading to reduced renal blood flow and impaired renal function (Barkin and Buvanendran, 2004; Harris, 2006). Further, our results confirm the results of former investigations that amelioration of cisplatin nephrotoxicity involves the inhibition of COX-II expression (Jia et al., 2011; Yu et al., 2017). This enhances the potential of COX-II enzyme as a therapeutic target for cisplatin-induced nephrotoxicity.

## Conclusion

Benzyl isothiocyanate exerted nearly comparable nephroprotective, antioxidant, and anti-inflammatory effects to RES. Moreover, the combination of both agents showed more potent protective effects than any agent alone. Further experimental and clinical investigations are required to confirm these findings and investigate their future therapeutic applications.

## Ethics Statement

All animal handling procedures in this study were approved by the Research Ethical Committee at the Faculty of Veterinary Medicine, Suez Canal University, Ismailia, Egypt (approval number: 201513).

## Author Contributions

AI, FA-H, and MA-D equally contributed to idea conceptualization, methodology, data curation, formal analysis, and writing of the manuscript. AIA contributed to methodology, formal analysis, and writing of the manuscript. All authors reviewed the final version of the manuscript and approved it for publication.

## Conflict of Interest Statement

The authors declare that the research was conducted in the absence of any commercial or financial relationships that could be construed as a potential conflict of interest.

## Abbreviations

BITC: benzyl isothiocyanate
CAT: catalase
COX-II: cyclooxygenase-II
GPx: glutathione peroxidase
GSH: reduced glutathione
IL-1β: interleukin-1β
MDA: malondialdehyde
RES: resveratrol
SOD: superoxide dismutase
TNF-α: tumor necrosis factor-α

## References

[B1] Aebi H. (1984). Catalase in vitro. *Methods Enzymol.* 105 121–126. 10.1016/S0076-6879(84)05016-3 6727660

[B2] Arany I. Safirstein R. L. (2003). Cisplatin nephrotoxicity. *Semin. Nephrol.* 23 460–464. 10.1016/S0270-9295(03)00089-5 13680535

[B3] Barkin R. L. Buvanendran A. (2004). Focus on the COX-1 and COX-2 agents: renal events of nonsteroidal and anti-inflammatory drugs-NSAIDs. *Am. J. Ther.* 11 124–129. 10.1097/00045391-200403000-00007 14999364

[B4] Beutler E. Duron O. Kelly B. M. (1963). Improved method for the determination of blood glutathione. *J. Lab. Clin. Med.* 61 882–888. 13967893

[B5] Catalgol B. Batirel S. Taga Y. Ozer N. K. (2012). Resveratrol: french paradox revisited. *Front. Pharmacol.* 3:141 10.3389/fphar.2012.00141 PMC3398412 22822401

[B6] Chou T. C. Talalay P. (1983). Analysis of combined drug effects: a new look at a very old problem. *Trends Pharmacol. Sci.* 4 450–454. 10.1186/s12889-014-1342-5 25884988 PMC4361135

[B7] Chou T. C. Talalay P. (1984). Quantitative analysis of dose-effect relationships: the combined effects of multiple drugs or enzyme inhibitors. *Adv. Enzym. Regul.* 22 27–55. 10.1016/0065-2571(84)90007-4 6382953

[B8] Coulombe J. J. Favreau L. (1963). A new simple semimicro method for colorimetric determination of urea. *Clin. Chem.* 9 102–108. 14023392

[B9] Dasari S. Tchounwou P. B. (2014). Cisplatin in cancer therapy: molecular mechanisms of action. *Eur. J. Pharmacol.* 740 364–378. 10.1016/j.ejphar.2014.07.025 25058905 PMC4146684

[B10] Do Amaral C. L. Della H. Francescato C. Machado T. Roberto C. Costa S. (2008). Resveratrol attenuates cisplatin-induced nephrotoxicity in rats. *Arch. Toxicol.* 82 363–370. 10.1007/s00204-007-0262-x 18026934

[B11] Duran-Salgado M. B. Rubio-Guerra A. F. (2014). Diabetic nephropathy and inflammation. *World J. Diabetes* 5 393–398. 10.4239/wjd.v5.i3.393 24936261 PMC4058744

[B12] Frémont L. (2000). Biological effects of resveratrol. *Life Sci.* 66 663–673. 10.1016/S0024-3205(99)00410-5 10680575

[B13] Galanski M. (2006). Recent developments in the field of anticancer platinum complexes. *Recent Pat. Anticancer Drug Discov.* 1 285–295. 10.2174/157489206777442287 18221042

[B14] Giovannini L. Migliori M. Longoni B. Das D. K. Bertelli A. Panichi V. (2001). Resveratrol, a polyphenol found in wine, reduces ischemia reperfusion injury in rat kidneys. *J. Cardiovasc. Pharmacol.* 37 262–270. 10.1097/00005344-200103000-00004 11243416

[B15] Goldstein R. S. Mayor G. H. (1983). The nephrotoxicity of cisplatin. *Life Sci.* 32 685–690. 10.1016/0024-3205(83)90299-0 6338333

[B16] Harris R. C. (2006). COX-2 and the kidney. *J. Cardiovasc. Pharmacol.* 47(Suppl. 1), S37–S42. 10.1097/00005344-200605001-00007 16785827

[B17] Hartmann J. T. Kollmannsberger C. Kanz L. Bokemeyer C. (1999). Platinum organ toxicity and possible prevention in patients with testicular cancer. *Int. J. Cancer* 83 866–869. 10.1002/(SICI)1097-0215(19991210)83:6<866::AID-IJC34>3.0.CO;2-9 10597214

[B18] Holthoff J. H. Wang Z. Seely K. A. Gokden N. Mayeux P. R. (2012). Resveratrol improves renal microcirculation, protects the tubular epithelium, and prolongs survival in a mouse model of sepsis-induced acute kidney injury. *Kidney Int.* 81 370–378. 10.1038/ki.2011.347 21975863 PMC3326404

[B19] Holthoff J. H. Woodling K. A. Doerge D. R. Burns S. T. Hinson J. A. Mayeux P. R. (2010). Resveratrol, a dietary polyphenolic phytoalexin, is a functional scavenger of peroxynitrite. *Biochem. Pharmacol.* 80 1260–1265. 10.1016/j.bcp.2010.06.027 20599800 PMC2934873

[B20] Jia Z. Wang N. Aoyagi T. Wang H. Liu H. Yang T. (2011). Amelioration of cisplatin nephrotoxicity by genetic or pharmacologic blockade of prostaglandin synthesis. *Kidney Int.* 79 77–88. 10.1038/ki.2010.331 20844471

[B21] Kim D. (2011). SIRT1 activation by resveratrol ameliorates cisplatin-induced renal injury through deacetylation of p53. *Am. J. Physiol. Renal Physiol.* 301 F427–F435. 10.1152/ajprenal.00258.2010 21593185

[B22] Kim M. Cho H. J. Kwon G. T. Kang Y. H. Kwon S. H. Her S. (2015). Benzyl isothiocyanate suppresses high-fat diet-stimulated mammary tumor progression via the alteration of tumor microenvironments in obesity-resistant BALB/c mice. *Mol. Carcinog.* 54 72–82. 10.1002/mc.22159 24729546

[B23] Kitada M. Kume S. Imaizumi N. Koya D. (2011). Resveratrol improves oxidative stress and protects against diabetic nephropathy through normalization of Mn-SOD dysfunction in AMPK/SIRT1-independent pathway. *Diabetes* 60 634–643. 10.2337/db10-0386 21270273 PMC3028365

[B24] Kruidering M. Van De Water B. De Heer E. Mulder G. J. Nagelkerke J. F. (1997). Cisplatin-induced nephrotoxicity in porcine proximal tubular cells: mitochondrial dysfunction by inhibition of complexes I to IV of the respiratory chain. *J. Pharmacol. Exp. Ther.* 280 638–649. 9023274

[B25] Larsen K. (1972). Creatinine assay in the presence of protein with LKB 8600 reaction rate analyser. *Clin. Chem. Acta* 38 475–476. 10.1016/0009-8981(72)90146-5 5026368

[B26] Lee Y. Kim Y. J. Choi Y. J. Lee J. W. Lee S. Chung H. W. (2012). Enhancement of cisplatin cytotoxicity by benzyl isothiocyanate in HL-60 cells. *Food Chem. Toxicol.* 50 2397–2406. 10.1016/j.fct.2012.04.014 22525867

[B27] Leonard S. S. Xia C. Jiang B. H. Stinefelt B. Klandorf H. Harris G. K. (2003). Resveratrol scavenges reactive oxygen species and effects radical-induced cellular responses. *Biochem. Biophys. Res. Commun.* 309 1017–1026. 10.1016/j.bbrc.2003.08.105 13679076

[B28] Miller R. P. Tadagavadi R. K. Ramesh G. Reeves W. B. (2010). Mechanisms of cisplatin nephrotoxicity. *Toxins* 2 2490–2518. 10.3390/toxins2112490 22069563 PMC3153174

[B29] Miyoshi N. Takabayashi S. Osawa T. Nakamura Y. (2004). Benzyl isothiocyanate inhibits excessive superoxide generation in inflammatory leukocytes: implication for prevention against inflammation-related carcinogenesis. *Carcinogenesis* 25 567–575. 10.1093/carcin/bgh051 14688023

[B30] Mokni M. Elkahoui S. Limam F. Amri M. Aouani E. (2007). Effect of resveratrol on antioxidant enzyme activities in the brain of healthy rat. *Neurochem. Res.* 32 981–987. 10.1007/s11064-006-9255-z 17401679

[B31] Morse M. A. Zu H. Galati A. J. Schmidt C. J. Stoner G. D. (1993). Dose-related inhibition by dietary phenethyl isothiocyanate of esophageal tumorigenesis and DNA methylation induced by N-nitrosomethylbenzylamine in rats. *Cancer Lett.* 72 103–110. 10.1016/0304-3835(93)90018-5 8402566

[B32] Nakamura Y. Miyoshi N. Takabayashi S. Osawa T. (2004). Benzyl isothiocyanate inhibits oxidative stress in mouse skin: involvement of attenuation of leukocyte infiltration. *Biofactors* 21 255–257. 10.1002/biof.552210149 15630206

[B33] Nishikimi M. Rao N. A. Yagi K. (1972). The occurrence of superoxide anion in the reaction of reduced phenazine methosulfate and molecular oxygen. *Biochem. Biophys. Res. Commun.* 46 849–854. 10.1016/S0006-291X(72)80218-3 4400444

[B34] Osman A. M. M. Telity S. A. Damanhouri Z. A. Al-Harthy S. E. Al-Kreathy H. M. Ramadan W. S. (2015). Chemosensitizing and nephroprotective effect of resveratrol in cisplatin–treated animals. *Cancer Cell Int.* 15:6. 10.1186/s12935-014-0152-2 25709558 PMC4337247

[B35] Paglia D. E. Valentine W. N. (1967). Studies on the quantitative and qualitative characterization of erythrocyte glutathione peroxidase. *J. Lab. Clin. Med.* 70 158–169. 6066618

[B36] Palsamy P. Subramanian S. (2011). Resveratrol protects diabetic kidney by attenuating hyperglycemia-mediated oxidative stress and renal inflammatory cytokines via Nrf2–Keap1 signaling. *Biochim. Biophys. Acta* 1812 719–731. 10.1016/j.bbadis.2011.03.008 21439372

[B37] Ramesh G. Reeves W. B. (2002). TNF-α mediates chemokine and cytokine expression and renal injury in cisplatin nephrotoxicity. *J. Clin. Invest.* 110 835–842. 10.1172/JCI200215606 12235115 PMC151130

[B38] Ramesh G. Reeves W. B. (2003). TNFR2-mediated apoptosis and necrosis in cisplatin-induced acute renal failure. *Am. J. Physiol. Renal Physiol.* 285 F610–F618. 10.1152/ajprenal.00101.2003 12865254

[B39] Ramesh G. Reeves W. B. (2004). Salicylate reduces cisplatin nephrotoxicity by inhibition of tumor necrosis factor-α. *Kidney Int.* 65 490–498. 10.1111/j.1523-1755.2004.00413.x 14717919

[B40] Robb E. L. Winkelmolen L. Visanji N. Brotchie J. Stuart J. A. (2008). Dietary resveratrol administration increases MnSOD expression and activity in mouse brain. *Biochem. Biophys. Res. Commun.* 372 254–259. 10.1016/j.bbrc.2008.05.028 18486604

[B41] Sastry J. Kellie S. J. (2005). Severe neurotoxicity, ototoxicity and nephrotoxicity following high-dose cisplatin and amifostine. *Pediatr. Hematol. Oncol.* 22 441–445. 10.1080/08880010590964381 16020136

[B42] Sharma S. Anjaneyulu M. Kulkarni S. Chopra K. (2006). Resveratrol, a polyphenolic phytoalexin, attenuates diabetic nephropathy in rats. *Pharmacology* 76 69–75. 10.1159/000089720 16286809

[B43] Siddik Z. H. (2003). Cisplatin: mode of cytotoxic action and molecular basis of resistance. *Oncogene* 22 7265–7279. 10.1038/sj.onc.1206933 14576837

[B44] Topcu-Tarladacalisir Y. Sapmaz-Metin M. Karaca T. (2016). Curcumin counteracts cisplatin-induced nephrotoxicity by preventing renal tubular cell apoptosis. *Ren. Fail.* 38 1741–1748. 10.1080/0886022X.2016.1229996 27758164

[B45] Uchiyama M. Mihara M. (1978). Determination of malonaldehyde precursor in tissues by thiobarbituric acid test. *Anal. Biochem.* 86 271–278. 10.1016/0003-2697(78)90342-1 655387

[B46] Ueki M. Ueno M. Morishita J. Maekawa N. (2013). Curcumin ameliorates cisplatin-induced nephrotoxicity by inhibiting renal inflammation in mice. *J. Biosci. Bioeng.* 115 547–551. 10.1016/j.jbiosc.2012.11.007 23245727

[B47] Valentovic M. A. Ball J. G. Brown J. M. Terneus M. V. Mcquade E. Meter S. V. (2014). Toxicology in Vitro Resveratrol attenuates cisplatin renal cortical cytotoxicity by modifying oxidative stress. *Toxicol. In Vitro* 28 248–257. 10.1016/j.tiv.2013.11.001 24239945 PMC3924893

[B48] Wattenberg L. W. (1987). Inhibitory effects of benzyl isothiocyanate administered shortly before diethylnitrosamine or benzo [a] pyrene on pulmonary and forestomach neoplasia in A/J mice. *Carcinogenesis* 8 1971–1973. 10.1093/carcin/8.12.1971 3677323

[B49] Yu X. Yang Y. Yuan H. Wu M. Li S. Gong W. (2017). Inhibition of COX-2/PGE2 cascade ameliorates cisplatin-induced mesangial cell apoptosis. *Am. J. Transl. Res.* 9 1222–1229. 28386348 PMC5376013

[B50] Yuan Y. Huang S. Wang W. Wang Y. Zhang P. Zhu C. (2012). Activation of peroxisome proliferator-activated receptor-γ coactivator 1α ameliorates mitochondrial dysfunction and protects podocytes from aldosterone-induced injury. *Kidney Int.* 82 771–789. 10.1038/ki.2012.188 22648295

[B51] Zhou Y. Chen K. He L. Xia Y. Dai W. Wang F. (2015). The protective effect of resveratrol on concanavalin-a-induced acute hepatic injury in mice. *Gastroenterol. Res. Pract.* 2015:506390. 10.1155/2015/506390 26089871 PMC4458299

